# Barriers and Enablers to Leadership in Advanced Practice Nursing: A Systematic Review

**DOI:** 10.1111/inr.70034

**Published:** 2025-05-22

**Authors:** Rosemary Fosah, Sofia Llahana

**Affiliations:** ^1^ University College London Hospital NHS Foundation Trust London UK; ^2^ School of Health and Medical Sciences, City St George's, University of London London UK

**Keywords:** Advanced practice nursing, barriers, enablers, leadership domain, leadership enactment, role capabilities

## Abstract

**Aim:**

To explore the barriers and enablers influencing leadership enactment by advanced practice nurses and assess how these have evolved over the past decade.

**Introduction:**

Nurses in advanced practice roles are well‐positioned to drive healthcare change, addressing patient needs and service demands. However, their leadership contributions are often underestimated, with greater emphasis placed on clinical practice and education rather than leadership and research.

**Methods:**

A systematic review of cross‐sectional studies was conducted using CINAHL, MEDLINE, EMBASE, APA PsycINFO, and Cochrane databases for studies published between January 2015 and March 2024. Fourteen studies involving 5243 participants were narratively synthesised. Study quality was assessed using the Joanna Briggs Institute Critical Appraisal Tool, and findings are reported following Preferred Reporting Items for Systematic Review and Meta‐Analyses (PRISMA) 2020 guidelines.

**Findings:**

The review identified 24 barriers and 18 enablers to enacting leadership in advanced practice nursing roles, grouped into eight themes across four structural levels: healthcare system, organisational, team, and individual. Key themes included leadership capacity building, role clarity, development opportunities, resource allocation, and mentorship. Major barriers were unclear roles, limited leadership training, and resource constraints, while enablers included mentorship, leadership programmes, and organisational support.

**Conclusion:**

Despite progress, significant barriers persist in developing leadership capabilities within advanced practice nursing roles, particularly in healthcare systems and organisational levels. Standardised education and training pathways are needed to equip nurses for leadership roles, and further research is required to address these barriers.

**Implications for nursing and health policy:**

Strengthening leadership capacity for advanced practitioners in nursing requires sustained institutional support, standardised education, and strategic engagement with policymakers. Maximising their leadership potential can drive healthcare innovation, improve patient outcomes, and ensure the long‐term sustainability of these roles.

**Trial and Protocol Registration:**

PROSPERO registration number CRD42024465777.

**Reporting Method:**

This systematic review adhered to relevant EQUATOR guidelines and followed the PRISMA guidelines.

## Introduction

1

### Evolvement and Definition of Advanced Practice Nursing

1.1

Advanced practice nursing is a globally recognised term describing nurses who practice at an advanced level, possessing specialist expertise beyond the generalist scope in a defined area of practice (Tracy et al. 2023). Advanced practice nurses (APNs) play a vital role in modern healthcare systems, particularly with the increased demand in complex procedures and medical interventions, contributing to enhanced patient‐care quality and the overall optimisation of population health worldwide (ICN 2020; Htay and Whitehead 2021; Unsworth et al. 2024). APNs help expand access to primary healthcare, particularly in rural communities, and address healthcare inequalities affecting vulnerable populations in urban areas. These contributions are essential to advancing universal health coverage and supporting the global effort to achieve the Sustainable Development Goals (SDGs) (WHO 2020). The APNs’ role development, responsibilities and capabilities may vary, depending on the level of country‐specific regulations, organisational readiness in practice, and educational standards (Lehwaldt et al. 2024; ICN 2020). Frameworks for advanced practice nursing exist to guide individual practitioners, promote a common understanding, support role delineation, and optimise role implementation and evaluation (Lehwaldt et al. 2024).

As defined by the International Council of Nurses (ICN), APNs acquire, through postgraduate education, an expert knowledge base in their specialty and demonstrate complex decision‐making skills and clinical competencies for expanded nursing practice (ICN 2020). Hamric and Hanson's model of advanced practice nursing further defines the core competencies essential for APNs, including direct clinical practice, expert coaching, consultation, research and evidence‐based practice, leadership, collaboration, and ethical decision‐making, all of which underpin advanced practice nursing across different healthcare systems, policies, and interprofessional collaboration (Tracy et al. 2023). In the United Kingdom, APNs operate under the definition of Advanced Clinical Practice set by Health Education England (HEE) in 2017 to ensure consistent, high‐quality healthcare standards across clinical settings (HEE 2017). This definition, consistent with earlier international definitions for advanced practice by the ICN and Hamric and Hanson (ICN 2020; Tracy et al. 2023), centres on four pillars: clinical practice, education, research, and leadership.

Working at this advanced level requires competencies in advanced clinical assessment, judgement, complex decision‐making, and autonomous diagnosing and prescribing. APNs serve as role models, demonstrating clinical expertise, professionalism, and autonomy (Tracy and Blumenthal 2023; Llahana et al. 2019). Empirical studies indicate that APNs enhance patient outcomes by providing consistent education and counselling (Woo et al. 2022) and contribute to staff and organisational improvements by reducing care fragmentation and promoting better care integration across professional boundaries (Duignan 2020). A systematic review on the effectiveness of APNs found that they have a positive impact on patient satisfaction, waiting times, chronic disease management, cost‐effectiveness, and overall quality of care (Htay and Whitehead 2021).

While advanced practice nursing roles are operational in more than 70 countries worldwide, they vary significantly across nations, influenced by political, regulatory, organisational, and local factors (Lamb et al. 2018; WHO 2020; ICN 2020; Kilpatrick et al. 2024). This was evidenced in a survey conducted by the ICN Advanced Practice Nursing Network in 18 countries almost two decades ago, which identified 13 different titles for APNs and highlighted role ambiguity as a major concern (Pulcini et al. 2010). However, a recent review confirms that role ambiguity for APNs remains a persistent issue, with the lack of standardised titles and definitions continuing to create confusion, hinder their integration into healthcare teams, and impact the clarity of advanced practice nursing responsibilities (Kilpatrick et al. 2024).

### The Leadership Domain of Advanced Practice Nursing

1.2

It can be argued that all nurses are leaders, with leadership being a shared responsibility across nursing roles. For APNs, leadership involves the autonomy to act, make independent decisions in patient care, and implement evidence‐based solutions to improve health outcomes and patient experiences (Reed and Carter 2023; ICN 2020; HEE 2017). Northouse (2018) defines leadership as the ability to influence a group towards a common goal. In healthcare, leadership is often embedded in complex care delivery processes. However, the leadership contributions of APNs are sometimes overlooked and may not be immediately evident to decision‐makers (Whitehead et al. 2017). While APNs prioritise clinical practice and education, leadership and research responsibilities often receive less emphasis within their role. This is reflected in self‐assessments using the Strong Model of Advanced Practice, where APNs rated their expertise highest in direct patient care and education but lower in research and leadership activities (Mick and Ackerman 2000).

Nevertheless, with their clinical expertise and master's‐level education, APNs are well‐positioned to lead and drive healthcare improvements, address patient needs and service demands, particularly in response to an ageing population and increasingly complex treatments (Reed and Carter 2023; Elliott 2017).

Understanding the barriers and enablers to APNs’ enacting leadership within their role, including their own perceptions of leadership, is crucial to maximising their potential and ensure the sustainability of these roles and optimise their impact on clinical practice, care delivery, and healthcare innovation. Leadership within these roles spans clinical, professional, systems, and health policy domains, often with substantial overlap (Reed and Carter 2023). Whilst the focus tends to be on developing clinical leadership first because of new clinical work, experienced APNs can demonstrate characteristics across all four domains (Reed and Carter 2023).

The SCAPE study in Ireland involving APNs and policymakers identified four key factors influencing APNs’ enacting of leadership: professional role development, access to leadership opportunities, mechanisms to sustain leadership, and personal attributes. APNs in solo roles with heavy clinical workloads had limited capacity for leadership, while management support was crucial in addressing professional isolation and expanding opportunities for APNs to influence policy and collaborate with other professionals (Begley et al. 2014; Higgins et al. 2014).

A systematic review by Elliott et al. (2016) explored the barriers and enablers to enacting leadership by APNs, identifying key factors across four structural layers: healthcare system level, organisational level, team level, and advanced practitioner level. Among the 13 identified barriers, most were linked to the organisational level, such as the lack of leadership capacity‐building strategies, gaps in leadership development, and heavy clinical workloads limiting time for leadership activities. Additionally, practitioner‐level factors, such as personal characteristics and educational background, played a significant role. Notably, these structural elements align with the leadership competencies outlined in Hamric and Hanson's Model of Advanced Practice Nursing, encompassing clinical, professional, health system, and health policy leadership (Tracy et al. 2023).

While Elliott et al. (2016) provided valuable insights into how APNs enact leadership, there is a need to reassess the empirical evidence in light of recent developments in advanced practice nursing following the publication of the ICN guidelines (ICN 2020). This underscores the ongoing importance of addressing systemic, organisational, and professional factors to optimise the leadership and effectiveness of APNs.

### Aim

1.3

This systematic review aimed to explore the barriers and enablers influencing APNs in incorporating leadership capabilities into their roles and to assess whether these factors have evolved since the Elliott et al. (2016) review.

## Methods

2

### Information Sources

2.1

The systematic review followed the Preferred Reporting Items for Systematic Reviews and Meta‐Analyses (PRISMA) guidelines (Page et al. 2021), and the protocol was registered with PROSPERO (registration ID: CRD42024465777). A preliminary search found a similar systematic review conducted by Elliott et al. (2016); therefore, the search was limited to any publications post‐January 2015. The initial search was completed on 14 January 2023 using the following databases: MEDLINE, EMBASE, EMCARE, CINAHL, APA PsychoINFO, and Cochrane. A full EMBASE search strategy is included in the Supplementary Material. A grey search and a secondary review of the reference lists of key articles were also undertaken to ensure all available evidence is reviewed. A secondary search, using the same strategy, was run covering the period between 15 January 2023 and 30 March 2024; no new studies were identified.

### Search Strategy

2.2

The objective of the systematic review informed the PICO framework which guided the search strategy comprising the following components:
Population: Nurses working in advanced practice rolesIntervention: Studies that examined the leadership domain within advanced practice nursing roles from the perspective of APNs enacting leadership capabilitiesComparator: Development in leadership capabilities for APNs since 2015Outcome measures: How APNs perform leadership capabilities


Search terms were hierarchically structured and combined with the Boolean operators (‘AND’, ‘OR’) of the following group keywords, truncations, and their respective synonyms and MeSH terms:

Population (APNs): ‘advanced nursing practice’ OR ‘advanced practice nursing’ ‘ANP’ OR ‘APN’ OR ‘advanced practice’ OR ‘advanced clinical practitioner’ OR ‘advanced practitioner’ OR ‘advanced nurse practitioner’ OR ‘nurse practitioner’ OR ‘clinical nurse specialist’ OR ‘registered nurse anaesthetist’ OR ‘nurse consultant’ OR’ clinical nurse consultant’.

AND

Intervention (Leadership): ‘leadership’ OR ‘clinical leadership’ OR leader* OR ‘professional leadership’ OR ‘manager’ OR ‘management’.

### Eligibility Criteria

2.3

#### Inclusion Criteria

2.3.1


The review included observational quantitative studies published after January 2015 that examined the leadership domain within advanced practice nursing roles. It focused on studies involving nurses working in roles classified under the umbrella of advanced practice nursing, as previously defined. Only peer‐reviewed studies published in English, involving human subjects and available in full text, were included.


#### Exclusion Criteria

2.3.2


The review excluded randomised controlled trials, comparative interventional studies, and qualitative research. It also excluded reviews, conference proceedings, editorials, opinion papers, and guidelines. Studies that focused primarily on role development or clinical outcomes were not considered. Additionally, manuscripts not published in English or those without full‐text availability were excluded.


### Selection Process

2.4

Records from the database search were exported to Rayyan, a software used to manage and collaborate on systematic reviews, and duplicates were removed. A two‐stage screening process was employed. The initial stage included screening the title and abstract of all records exported onto Rayyan by the first author (RF); the second author (SL) blind‐screened 40% of the abstracts for validation purposes. In the second stage, the retrieved full‐text papers were scrutinised against the inclusion criteria independently by both authors, with a 90% agreement level, and any discrepancies were resolved between the authors. It was not deemed necessary to involve a third researcher.

### Data Collection Process

2.5

An Excel data collection spreadsheet was used to extract data from each study; the second author contributed to the data extraction for five randomly selected studies to ensure uniformity and agreement with the data extraction process.

### Data Items and Outcome Measures

2.6

For each of the included studies, the following information was recorded: (1) country and study setting, (2) sample and data collection measures, and (3) outcomes relating to factors that influence APNs from enacting their leadership capabilities. Only findings related to the leadership aspect of the role were extracted, including mean and standard deviation where applicable, with reported *P* values of less than 0.05 indicating statistical significance.

### Study Risk of Bias Assessment

2.7

The quality appraisal was undertaken to aid the interpretation of findings and to assist in determining the strength of the conclusions drawn. The Joanna Briggs Institute (JBI) Critical Appraisal Tool for analytical cross‐sectional studies (Moola et al. 2015) was used to appraise the studies to ensure validity and to examine the reliability with a quantifiable score on each included study. Each question was dichotomised to either Yes (1 point) or No (0 points) producing a scale where if studies had five or fewer ‘Yes’ points, they were categorised as ‘poor quality’, six or seven ‘Yes’ points as ‘fair quality’ and all eight ‘Yes’ points as ‘good quality’. Risk of bias was assessed independently by two authors (RF and SL), following the same process as used for screening. Both reviewers evaluated all included studies, and any disagreements were resolved through discussion until a consensus was reached. No formal adjudication was required as full agreement was achieved for all included studies (Table 1).

### Synthesis Methods

2.8

A narrative synthesis and descriptive analysis were conducted due to the heterogeneity of the included studies. The synthesis focused on identifying factors influencing APNs in enacting leadership within their roles. To ensure rigour and transparency, an inductive approach was applied, with two authors (RF and SL) independently extracting and coding data to identify key patterns. Thematic groupings were initially guided by emerging themes and informed by leadership models within advanced practice nursing, particularly the framework outlined by the earlier systematic review by Elliott et al. (2016). The identified factors were categorised into ‘barriers’ and ‘enablers’ and mapped onto the four structural levels proposed by Elliott et al. (2016): healthcare system, organisational, team, and advanced practitioner levels. To enhance reliability, the themes were reviewed, refined, and agreed upon through consensus discussions, ensuring consistency in interpretation. The final themes were validated against the study objectives to ensure their relevance and coherence within the broader leadership context in advanced practice nursing roles.

## Findings

3

### Study Selection and Characteristics

3.1

**FIGURE 1 inr70034-fig-0001:**
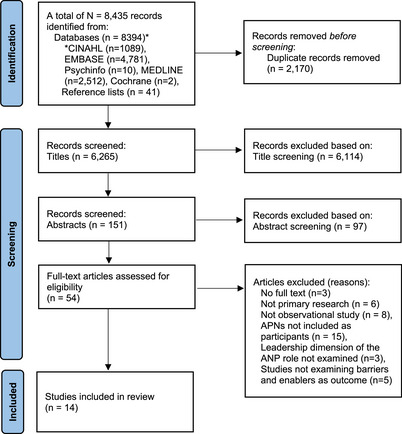
PRISMA Flowchart of study search, screening, and selection.

A total of 8435 records were extracted from the initial search, and 2170 duplicate records were removed, resulting in 6265 records. The first stage of screening eliminated 6114 records at title screening and 97 at abstract screening. A total of 54 full‐text articles were retrieved for screening against the eligibility criteria, of which 40 were excluded, giving a final number of 14 studies that met the inclusion criteria and were included in the narrative synthesis. A detailed summary of the screening is presented in the PRISMA Flowchart in Figure 1.

The studies were conducted across various countries in Europe, Asia, Oceania, and the United States. Sample sizes ranged from 32 to 4014 participants, with a total of at least 5243 participants. However, two studies did not specify the number of APNs within the total participant pool. In these cases, it was assumed the findings applied equally to the APN subset. Sampling generally targeted existing lists of registered APNs within professional groups or organisations or relied on self‐identification due to the unregulated nature of the title, making participant identification challenging (Table 2).

**TABLE 1 inr70034-tbl-0001:** Characteristics of included studies and outcomes related to leadership capabilities in advanced practice nursing.

Author and year	Country and setting	Sample	Tools and measurements	Study outcomes	JBI score
Denker et al. (2015)	Florida State, USA Statewide	Nurses in formal leadership roles (*N* = 1354) ‘*n*’ of ARNP not specified 90% female overall 39% worked in ACH 61% Variety community‐based/academic settings 45% Graduate level (inc MLP)	Online self‐completed survey Measurements for leadership capabilities: 1. Closed questions identifying key leadership competencies 2. Rank top competencies from the list provided 3. Identify perceived barriers to leadership from the list provided	87% of respondents were confident in their knowledge and 92% in practice of nursing leadership. 89% had participated in leadership programmes to gain knowledge and skills. 56% either had been mentored or were mentoring others.	F
Fothergill et al. (2022)	England Nationwide	ACP staff *N* = 4013 66.5% female *n* = 186 from PCOs *n* = 166 NHS providers 56.9% MLP	Online self‐completed survey—mixed quantitative and qualitative items: 1. Binary questions (yes/no) on governance structures, education attainment, level of supervision and support 2. Free text on the experience of mentoring support 3. Likert‐style scale question on knowledge of the HEE framework	Widely held belief amongst ACPs that four pillars of AP integral to ACP roles. Clinical practice pillar consistently prioritised over 3 remaining pillars, attributed to the demanding nature of the role. 37% of ACP respondents undertaking master's level training. Poor knowledge of the HEE framework amongst respondents (36%) in primary care.	F
Ryder et al. (2020)	Ireland Australia Nationwide	NPs (N = 96) *n* = 22 from Ireland; *n* = 74 from Australia 80% Female overall 28% worked in ED 74% at least MLP	Online self‐completed survey 1. Scale (0–10) to measure quantity of leadership within a role 2. Likert‐style scale on leadership activities and outcomes	15% M (SD 14) of NP time dedicated to administration and management compared to 54% (SD 27) on direct patient care. Majority of NPs perceived providing strong leadership M 7.5 out of 10 (SD 2.17) [Ireland 7.23 (SD 1.9); Australia 7.59 (SD 2.3, *p* = 0.47]. Perception of providing strong leadership increased across both countries with age (Australia: M 7.53 {SD 2.50} for 25–44 years category compared to M 9.00 {SD 0.00} for 65+ years category) and years practised as NP (Australia M 7.65 {SD 1.96} for 0–5 years category vs M 8.00 {SD 2.75) for 11–15 years category. Research‐active NPs (58%) reported a higher leadership score (M 8, SD 1.8) than non‐research–active NPs (42%) with M 7(SD 2.5, *p* = 0.02).	F
Sevilla Guerra et al. (2018)	Spain Hospitals	*N* = 151 inc. SNs and ENs (*n* = 115) 15.2% worked in OP, 6.6% in ED 56% at least MLP	Online self‐completed survey generated using the Spanish version of the APRD tool: Likert scale (0–4) to measure time spent across six practice domains including interprofessional collaboration, education, and professional leadership	Nursing grade on the professional ladder related to four of the six APN domains including professional leadership (*p* < 0.001). Nurses on lower levels of the professional ladder performed fewer APN activities in the leadership domain. Age (*p <* 0.001) and YoE (*p <* 0.001) have an association with professional leadership; in particular >15 YoE had a predictive impact on professional leadership (β coefficient 0.7 {0.2}, *P* = 0.002). Higher levels of education (master's degree/PhD) were associated with more professional leadership (*p* = 0.004).	G
van Hecke et al. 2019)	Flanders, Belgium Peripheral and university hospitals	*N* = 63 APNs (*n* = 58) 87.3% female overall 100% Hospital‐based 100% MLP	Online self‐completed survey Inventory of APN competencies and task performance (based on domains by Hamric et al. 2013) measured using: 1. Battery of binary questions (yes/no) whether task carried out 2. Likert‐style scale for what extent they felt competent with carrying out the task	Participants focused on guideline and care protocol development within the hospital (95%); extending and maintaining contacts with APs in other POTS (85%); and participating in policy development meetings regarding domain‐specific topics (81.7%). 36.7% of APNs participated in policy meetings at the hospital and 43.3% at the departmental level, although in each instance, 62.1% and 67.2%, respectively, felt competent to do so. Similar figures and patterns were found for participation in national and international advisory boards. Association found between APNs not feeling competent and non‐participation in policy meetings at hospital/departmental level (OR 34.80, 95% CI 3.64–332.54).	G
Stewart et al. (2018)	England, UK NHS Hospital Trust	LCNS *N* = 230	Online self‐completed survey Inventory of UK‐context‐specific questions to measure workload, working practices and leadership activities for LCNSs	77.8% of LCNS had active MDT attendance, yet only 51.7% would challenge any MDT members. LCNS at hospitals with high caseloads were more likely to challenge any MDT members (*p* = 0.0007) compared to those from lower caseload trusts.	G
Jokiniemi et al. (2022)	Hong Kong In clinical role	Inc NCs and APNs *N* = 140 73.5% and 80.2% female in NC and APN, respectively. 88.2% and 84% in ACH for NC and APN, respectively. Min 85.3% and 92.5% MLP in NC and APN, respectively.	Online self‐completed survey APRD tool used to measure practice activities across five domains (Strong Model) and perception of time spent overall during a typical month in each domain: 5‐point Likert ‐type scale used	NCs were most active, with the highest involvement in publication and leadership domain activities than other groups. APNs spent the least amount of time in the publication and professional leadership domain.	G
McCarthy et al. (2019)	Republic of Ireland Nationwide	All grades nurses and midwives (*N* = 321) *n* = 32 AP 91% female overall 23% worked in general; 12% community; 3% PCO 23% MLP	Online‐ and postal self‐completed survey Measured importance of leadership in clinical practice and performance of leadership: Used 7‐point Likert‐type scale	Mean score for the perceived importance of leadership and clinical practice dimension is higher than the score for actual performance 77.1/100 (SD 7.8) vs 51.9 (SD 14.5).	G
Williams and Li (2019)	North Carolina USA Hospital setting	RNs and APRNs (in formal leadership roles) *N* = 1286 *n* = APRNS not specified 93.7% female overall 33% in hospital setting	Online self‐completed survey Measured leadership experience, reasons for success as nurse leaders, barriers to leadership opportunities and development needs using: Combination of Likert‐type scale and ranked options from the list provided	More than half of respondents identified that the scope and responsibilities of the current role needed to be re‐examined (81%). Top 3 competencies for nurse leader success: communication (44.9%), knowledge of the healthcare environment (19.5%), and clinical experience (10.03%).	F
Poghosyan and Bernhardt (2017)	New York USA Primary care	NPs *N* = 278 90.2% female 53% in physician office setting; 34% hospital‐affiliated 84% MLP	Online self‐completed survey Measured conceptual and empirical factors of transformation leadership from the perspective of the NP using Likert‐type questions	33.8% of NP did not receive performance feedback. >45% of NPs reported they were not represented on important organisational committees. 40% of NPs not encouraged by their administration to share their ideas. ∼40% of NPs reported not being listened to by the organisational leadership. >35% of NPs responded that their leadership did not support their ideas or pay attention to NP's request.	G
Fernandez et al. (2017)	New South Wales Australia Metropolitan Health District	*N* = 122 Inc CNCs *n* = not specified 90% female 100% hospital setting 69% min MLP	Online self‐completed survey Measured key drivers and mitigating factors to role performance using Likert‐type questions	Key driver impacting role identified as personal attribute—personal motivation and own communication skills—M 7.7 / 8 (SD 0.6). Second driver was experience—clinical experience and own understanding of role—M 7.5 / 8 (SD 0.9). Other drivers included peer support (M 9.3 / 12, SD 2.1), professional learning (mean 6.6 / 8, SD 1.1), collaborative relationship (mean 7.2 / 8, SD 0.9), and organisational support (M 10.2 / 12, SD 1.8). Mitigating factors identified as: lack of resources (M 2.6, SD 0.6), lack of secretarial support (M 2.6, SD 1.1), lack of CPD (M 2.3, SD 1.0), lack of clinical support (M 2.2, SD 1.0), lack of managerial support (M 212.4, SD 1.1), and lack of academic qualification as lowest at 1.6.	F
East et al. (2015)	East Midlands UK NHS Trust	ANPs, CNS, NPs, and NCs *N* = 136 No information on gender 100% within ACH *n* = 23 MLP	Online self‐completed survey Measured: ‐ Activities undertaken within APN roles using closed‐option questions ‐ Achievements and future development needs using free‐text questions	Estimated 67% of participants’ time was spent in clinical practice compared to 15 % on management and administration; 4% on service development and 4% on CPD. 96% of respondents identified one or more training needs. 38% of respondents reported making considerable contributions at local, regional, and national levels.	F
Woo et al. (2019)	Singapore 67.8% in acute care	APNs *N* = 87 93% female 68% ACH 95% MLP	Online self‐completed survey Measured: APN practice patterns across 5 domains (Strong Model) and perspectives/attitudes towards APN role expansion using Likert‐style questions.	Prominent difference in the time participants expected to spend and current practice observed in the professional leadership domain (*p* < 0.001). Participants spent the least amount of time (<30%) and rated themselves worse in ‘research’ and ‘publication and professional leadership’ (median 6, IQR 5–7).	G
Florez et al. (2022)	Cincinnati, USA Paediatric CICU	*N* = 107 Inc APPs (*n* = 53) 100% ACH No information on Gender, MLP	Online self‐completed survey Measured APP emergency leadership education and practice activities using a combination of closed question (yes/no) and Likert‐style question	50.9% of APPs felt supported by attending physicians in leading codes/other emergencies	P

Abbreviations: ACH, Acute Care Hospital; ACP, Advanced Clinical Practitioner; APs, Advanced Practitioners; ANPs, Advanced Nurse Practitioners; APNs, Advanced Practice Nurses; APPs, Advanced Practice Providers; ARNPs, Advanced Registered Nurse Practitioners; CI, Confidence Interval; CPD, Continuing Professional Development; CICU, Cardiac Intensive Care Unit; CNCs, Clinical Nurse Consultants; CNSs, Clinical Nurse Specialists; ED, Emergency Department; ENs, Expert Nurses (nurses with a higher practice profile and more extended service profile than general nurses; F, fair quality; G, good quality; JBI, Joanna Briggs Institute Assessment of Bias Score; LCNS, Lung Cancer Nurse Specialists; M, Mean score; MDT, Multidisciplinary Team; MLP, Masters Level Prepared; NPs, Nurse Practitioners; NCs, Nurse Consultants; OR, Odd Ratio; P, poor quality; PCOs, Primary Care Organisations; POTs, Provider Organisations/Trusts; RNs, Registered Nurses; SD, Standard Deviation; SNs, Specialist Nurses; YoE, Years of Experience.

**TABLE 2 inr70034-tbl-0002:** Barriers and enablers to enacting leadership in advanced practice nursing roles.

Structural layer	Overall themes	Barriers to leadership enactment	Enablers to leadership enactment
Healthcare system level	General perception of nursing roles	(*N* = 1699) Public perception of nursing roles (Denker et al. 2015; Williams and Li 2019; Woo et al. 2019) (*N* = 1924) Nurses not viewed as major revenue generators compared to physicians (Denker et al. 2015; Williams and Li 2019) (*N* = 1625) Inadequate financial compensation for nurses (Denker et al. 2015; Williams and Li 2019) Lack of standardised national policy (Fothergill et al. 2022)	
	‘Sit at the table of power’ strategic policy‐making level	(*N* = 1740) Absence of nurse visibility in policy‐making at the strategic level (Denker et al. 2015; Williams and Li 2019)	(*N* = 351) Clear vision (Denker et al. 2015; Woo et al. 2019)
	Training/educational preparation policies		(*N* = 1114) Educational preparation Denker et al. 2015; Williams and Li 2019) Supportive regulatory environment; APN involvement in the development of national policies (Woo et al. 2019) (*N* = 192) Opportunities for guideline/quality criteria and policy development within the organisation (van Hecke et al. 2019)
Organisational level	Resources, time, and workload	(*N* = 754) Lack of resources and funding (Denker et al. 2015; Fothergill et al. 2022; Fernandez et al. 2017; East et al. 2015; Woo et al. 2019) Lack of infrastructure—clinic space (Woo et al. 2019) Lack of protected study time (Fothergill et al. 2022; East et al. 2015; Woo et al. 2019) Large workloads (Fothergill et al. 2022; van Hecke et al. 2019; Woo et al. 2019) Competing non‐clinical duties (Woo et al. 2019; Fothergill et al. 2022)	
	Supervision, mentoring, and support	Inadequate/inconsistent levels of supervision (Fothergill et al. 2022; Woo et al. 2019) Lack of clinical and organisational leadership/management support (Fernandez et al. 2017; Fothergill et al. 2022; Woo et al. 2019; Poghosyan and Bernhardt 2017) (*N* = 466) Lack of administrative support (van Hecke et al. 2019; Fernandez et al. 2017; Poghosyan and Bernhardt 2017)	(*N* = 678) Mentoring (Denker et al. 2015; Williams and Li 2019; Woo et al. 2019; Fothergill et al. 2022; Florez et al. 2022) Organisational Support (Fothergill et al. 2022; Woo et al. 2019; Fernandez et al. 2017)
	Commitment to leadership capacity building	(*N* = 122) Lack of representation on important organisational committees (Poghosyan and Bernhardt 2017) (*N* = 94) Lack of performance feedback/CPD support (Poghosyan and Bernhardt 2017; Fernandez et al. 2017)	Support APN development: CPD, greater clinical exposure (Woo et al. 2019; Fernandez et al. 2017)
	Clarity and understanding of role	Institutional regulatory environment such as poorly defined job scope (Woo et al. 2019) Inconsistencies in ACP titles and role (Fothergill et al. 2022) Lack of acceptance from physicians, nursing leaders and at the institutional level (Woo et al. 2019)	Regulatory environment: policies that clearly defined job scope (Woo et al. 2019) General acceptance of APNs (Woo et al. 2019)
	Development opportunities, programmes and training		Networking and other collaborative relationships (van Hecke et al. 2019; Williams and Li 2019; Fernandez et al. 2017) (*N* = 759) Leadership opportunities/participation (Denker et al. 2015; Fernandez et al. 2017; Florez et al. 2022; Williams and Li 2019) (*N* = 457) Leadership training and development programmes—formal and informal (Denker et al. 2015; McCarthy et al. 2019; Williams and Li 2019; East et al. 2015; Woo et al. 2019; Florez et al. 2022 (*N* = 4,187) Educational opportunities (Fothergill et al. 2022; Sevilla Guerra et al. 2018; Williams and Li 2019)
Team level	Commitment to leadership capacity building	Lone APN position impact on undertaking CPD (East et al. 2015)	
	Development opportunities, programmes, and training		Peer support and other collaborative relationships (Fernandez et al. 2017)
	Clarity and understanding of role	Lack of recognition of APN role within the wider team (Fothergill et al. 2022)	
Advanced nurse practitioner level	Commitment to leadership capacity building	Personal attributes: lack of self‐confidence (van Hecke et al. 2019; Stewart et al. 2018) (*N* = 254) Personal characteristics: gender and ethnicity (Williams and Li 2019)	Personal attributes: self‐motivation and communication skills (Fernandez et al. 2017; Williams and Li 2019) Personal characteristics: Age (Ryder et al. 2020; Sevilla Guerra et al. 2018; Jokiniemi et al. 2022)
	Development opportunities, programmes, and training	*N* = 311) Level of education (Williams and Li 2019; Fernandez et al. 2017)	Level of education (Sevilla Guerra et al. 2018; Jokiniemi et al. 2022)
	Clarity and understanding of the role		(*N* = 1015) Time in role/experience (Fernandez et al. 2017; Jokiniemi et al. 2022; Sevilla Guerra et al. 2018; Williams and Li 2019; Ryder et al. 2020; Denker et al. 2015)

All studies employed an observational cross‐sectional quantitative methodology using online or postal survey for data collection. Table 1 summarises the primary outcomes related to leadership, such as leadership knowledge, skills, and activities, along with secondary outcomes related to independent factors influencing leadership.

### Critical Appraisal

3.2

Seven out of 14 studies were of good quality, as they clearly explained their analytical strategies and provided relevant data to address the study question (Sevilla Guerra et al. 2018; Poghosyan and Bernhardt 2017; Stewart et al. 2018; McCarthy et al. 2019; van Hecke et al. 2019; Woo et al. 2019; Jokiniemi et al. 2022). Six studies were of fair quality, mainly due to insufficient descriptions of the study setting and subjects (Denker et al. 2015; East et al. 2015; Fernandez et al. 2017; Williams and Li 2019; Ryder et al. 2020; Fothergill et al. 2022). One study was rated poor quality due to limitations in reporting outcome measures and confounding factors (Florez et al. 2022). None of the studies were excluded based on the results of the quality assessment as per JBI recommendations (Supplementary Material).

### Results of Synthesis

3.3

**FIGURE 2 inr70034-fig-0002:**
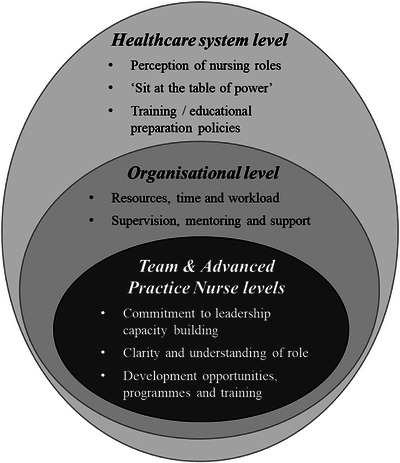
Model of factors influencing leadership enactment in advanced practice nursing roles.

The narrative synthesis identified 24 barriers and 18 enablers, grouped into eight overarching themes and categorised across four structural levels: (1) healthcare system, (2) organisational, (3) team, and (4) APNs (Figure 2).

Some factors either supported or constrained the enactment of leadership, depending on their presence or absence. Additionally, there was an overlap in themes across the four structural levels (as shown in Table 2), indicating that certain factors influencing leadership can operate both internally at the APN level and externally beyond the APN's control. The eight overarching themes were:
Commitment to leadership capacity buildingClarity and understanding of the roleDevelopment opportunities, programmes, and trainingResources, time, and workloadSupervision, mentoring, and supportGeneral perception of nursing roles‘Sit at the table of power’Training/educational preparation policies


As delineated in Table 2, the *organisational level* was the largest category, covering six themes (14 barriers and 10 enablers). This was followed by the *healthcare system level* with three themes (5 barriers and 3 enablers), the *advanced practitioner level* with three themes (3 barriers and 4 enablers), and the *team level* with three themes (2 barriers and 1 enabler).

### Commitment to Leadership Capacity Building

3.4

Five barriers were identified within this theme, with three occurring at the organisational level. Two studies noted that APNs lacked performance feedback and continuing professional development support, both of which are key aspects of inspirational motivation and are essential for goal attainment when provided (Poghosyan and Bernhardt 2017; Fernandez et al. 2017). Additionally, the lack of APN representation on organisational committees was reported as another significant barrier (Poghosyan and Bernhardt 2017).

At a team level, one study recognised the impact of the lone APN position as inhibiting their ability to undertake continuing professional development and in turn leadership capacity building (East et al. 2015). Personal attributes were identified as a barrier to leadership which related to a lack of confidence to participate in leadership activities; ‘not feeling competent and intimidated’ was a common barrier which prevented APNs from participating in multidisciplinary and policy meetings (van Hecke et al. 2019; Stewart et al. 2018). Another study highlighted that APNs felt being overlooked due to personal characteristics such as age, gender, and ethnicity (Williams and Li 2019). Three enabling factors were identified within this theme. Support for APN development such as continuous professional development and greater clinical exposure were identified as enablers at an organisational level (Woo et al. 2019; Fernandez et al. 2017), and personal attributes such as self‐motivation and communication skills as enablers at the APN level (Fernandez et al. 2017; Williams and Li 2019). Moreover, APNs’ perception of providing strong leadership increased with age and experience in the role (Ryder et al. 2020; Sevilla Guerra et al. 2018; Jokiniemi et al. 2022).

### Clarity and Understanding of Role

3.5

This theme was particularly relevant at the organisational level, where three of the four barriers were identified. These included a lack of acceptance from physicians, nursing leaders, and employers (Woo et al. 2019), as well as inconsistencies in the titles and job descriptions across advanced practice nursing roles (Fothergill et al. 2022). Woo et al. (2019) also noted that institutional regulatory environments could act as a barrier to APNs enacting leadership when their job scope was poorly defined, but served as an enabler when the job scope was clearly defined, leading to greater acceptance of the APN role. Similarly, at the team level, role inconsistencies contributed to a lack of recognition of the APN role within the wider team, resulting in frustration (Fothergill et al. 2022). Experience as an APN and time spent in the role were identified as enablers at the APN level in six studies (Fernandez et al. 2017; Jokiniemi et al. 2022; Sevilla Guerra et al. 2018; Williams and Li 2019; Ryder et al. 2020; Denker et al. 2015).

### Development Opportunities, Programmes, and Training

3.6

Although the factors under this theme primarily served as enablers, the level of education of the APN either acted as a barrier when inadequate (Williams and Li 2019; Fernandez et al. 2017) or as an enabler for those with higher levels of education (Sevilla Guerra et al. 2018; Jokiniemi et al. 2022). Additionally, six enablers were identified under this theme. At the organisational level, a significant number of participants highlighted educational opportunities in three studies (Fothergill et al. 2022; Sevilla Guerra et al. 2018; Williams and Li 2019), followed by leadership opportunities and participation (Denker et al. 2015; Fernandez et al. 2017; Florez et al. 2022; Williams and Li 2019), as well as leadership training and both formal and informal development programmes (Denker et al. 2015; McCarthy et al. 2019; Williams and Li 2019; East et al. 2015; Woo et al. 2019; Florez et al. 2022). Lastly, networking and other collaborative relationships to extend and maintain contacts with other APNs were reported as essential for enacting leadership roles (van Hecke et al. 2019; Williams and Li 2019; Fernandez et al. 2017). Some overlap was noted, particularly in collaborative relationships and peer support at the team level (Fernandez et al. 2017).

### Resources, Time, and Workload

3.7

Five barriers were identified in this theme with lack of resources and funding being the most frequently reported (Denker et al. 2015; Fernandez et al. 2017; East et al. 2015; Woo et al. 2019; Fothergill et al. 2022). Resources were not readily available to support APNs’ leadership activities (Denker et al. 2015) and one in four APNs made personal financial contributions to their studies, with lack of funding perceived to have restricted role performance (Fothergill et al. 2022). Other barriers included a lack of protected study time resulting in APNs feeling overwhelmed by the volume of academic work alongside their large clinical workload, competing non‐clinical duties, and lack of infrastructure to support APN services such as clinic space (van Hecke et al. 2019; Woo et al. 2019; Fothergill et al. 2022). These barriers had a negative impact on available time for non‐clinical leadership activities, restricted APNs’ role performance and limited their opportunities to take on leadership.

### Supervision, Mentoring, and Support

3.8

Organisational and management support was identified as an enabler to APNs’ leadership enactment, while the lack of it as a barrier (Fernandez et al. 2017; Poghosyan and Bernhardt 2017; Woo et al. 2019; Fothergill et al. 2022). Woo et al. (2019) also reported that some APNs felt that support was lacking not only at an institutional level but also from physicians and nursing leaders. General acceptance of APNs was deemed facilitative including trust and support from key stakeholders such as patients, physicians, nurses, and management (Woo et al. 2019). Lack of administrative support (Fernandez et al. 2017) and inadequate or inconsistent levels of clinical supervision were additional barriers (Woo et al. 2019; Fothergill et al. 2022), with only 32% of APNs having formal structures for supervision in place (Fothergill et al. 2022). On the contrary, mentorship was one of the most frequently reported enablers and was ranked as one of the top three reasons for leadership success (Denker et al. 2015; Williams and Li 2019; Woo et al. 2019; Fothergill et al. 2022; Florez et al. 2022).

### General Perception of Nursing Roles

3.9

At the healthcare system level, a significant barrier to APNs attaining leadership roles was the general perception that nurses, unlike physicians, are not major revenue generators. This may be due to the value placed on nursing, which is reflected in healthcare payment and reimbursement systems (Denker et al. 2015; Williams and Li 2019). Additional barriers included inadequate financial compensation for APNs and the limited understanding of APN roles among the wider public (Denker et al. 2015; Williams and Li 2019). Participants also noted that the negative perception of APNs among nurses themselves and the lack of trust from other healthcare professionals, potentially due to a poor understanding of the role, hindered their practice and, consequently, their ability to enact leadership (Woo et al. 2019).

### ‘Sit at the Table of Power’

3.10

This theme highlights the importance of visibility in management structures at the executive or director level, which affects an APN's ability to influence strategic decision‐making and provide leadership in healthcare service development. The absence of such visibility was identified as a barrier at the system level (Denker et al. 2015; Williams and Li 2019). Conversely, having a clear vision, professional influence, and visibility at this level empowered APNs to fully utilise their leadership training and engage in national policy, promoting greater recognition and awareness of APN roles (Denker et al. 2015; Woo et al. 2019).

### Training/Educational Preparation Policies

3.11

The lack of a standardised national policy to guide advanced practice nursing governance structures, developmental pathways, and career progression, particularly concerning guidance, supervision, and support, was identified as a barrier at the healthcare system level (Fothergill et al. 2022). This led to a perceived lack of direction and concerns about the future sustainability of the APN role (Fothergill et al. 2022). Conversely, educational preparation (Denker et al. 2015; Williams and Li 2019), a supportive regulatory environment with policies favouring greater autonomy in patient care (Woo et al. 2019), and opportunities for APNs to contribute to the development of guidelines, quality criteria, and policies within their organisations (van Hecke et al. 2019) were reported as enablers of leadership enactment.

## Discussion

4

### Summary of Findings

4.1

This systematic review identified 24 barriers and 18 enablers to APN leadership, grouped into eight themes across four structural levels. Hospitals are often complex sociopolitical environments where leadership is hindered by power dynamics, disciplinary boundaries, and competing discourses with healthcare organisations (Daly et al. 2014), reflecting key findings of this review and reporting similar barriers and enablers to those in the Elliott et al. (2016) review. However, this systematic review suggests that factors influencing leadership can be both internal (at the APNs’ level) and external (beyond APNs' control), introducing new dimensions: commitment to leadership capacity building and perceptions of nursing roles. This is significant as it provides up‐to‐date evidence reflecting changes following the publication of the guidelines for Advanced Practice Nursing by the International Council of Nursing (ICN 2020).

### Healthcare System Level

4.2

An important finding from this review was the increase in barriers attributed to the healthcare system level, with most relating to the theme of the ‘general perception of nursing roles’. Greater awareness and education of the public and medical professionals on the APNs’ role within the healthcare team that nurses operate at different levels of expertise, providing tailored services to specific patient groups, would facilitate public support, thereby aiding role development and implementation (Casey et al. 2019). Clear communication of the APNs’ role within and outside of the profession, with well‐defined leadership responsibilities supported at a strategic level, is essential for enabling the full operationalisation of the APNs’ capabilities. This is particularly relevant as many countries are at different stages of developing advanced practice nursing roles within the nursing workforce (ICN 2020).

For instance, a recent nationwide evaluation of the ACP role in England revealed a lack of clarity among stakeholders regarding role definition, preparation, and scope of practice, impacting implementation and sustainability (Evans et al. 2021). This was linked to varying competencies and educational backgrounds among APNs (Evans et al. 2021), a challenge also noted in an international review which showed a lack of awareness, understanding, and clarity of the advanced practice nursing roles (Mackavey et al. 2024). These findings highlight the urgent need for standardised minimum master's‐level education and training pathways, alongside sector‐specific training as per international guidelines for advanced practice nursing (ICN 2020). Furthermore, a supportive regulatory environment, with accreditation frameworks for education and preparatory training, alongside leadership opportunities, is essential for developing the right cadre of APNs (Fealy et al. 2018). The World Health Organisation emphasises that modernisation is imperative within professional nursing regulation; it includes harmonisation and mutual recognition of nursing education, credential standards, and professional credentials plus interoperable systems that allow regulators to verify nurses’ credentials (WHO 2020).

### Organisational Level

4.3

The organisational level remained the dominant category, aligning with findings from Higgins et al. (2014) and Elliott et al. (2016), which highlight gaps in factors such as strategic positions, leadership capacity building, and role clarity which influence leadership enactment. Within its top ten key actions for the future direction for nursing workforce policy, the World Health Organisation emphasises building capacity to nurture nursing leadership development and governance (WHO 2020). Strengthening nurse leadership would ensure a *‘Sit at the table of power*’ and an influential role in health policy creation and decision‐making and contribute to the effectiveness of health and social care systems that all steer towards the agenda for universal health coverage (WHO 2020). APNs are increasingly recognised as well‐positioned with unique knowledge and clinical legitimacy that provides them a strong platform for leadership (Reed and Carter 2023) within healthcare organisations, which should focus their strategic plans on targeted leadership development (Elliott 2017). The ‘*Future of Nursing 2020–2030: Charting a Path to Achieve Health Equity’* report emphasised the necessity for robust nursing leadership at all levels, both within the profession and in interprofessional settings, to collaboratively transform health systems (National Academies of Sciences, Engineering, and Medicine 2021).

Given the multi‐professional nature of healthcare organisations, the process of leadership capacity building remains multifaceted and complex. As such, organisations should clarify and promote the APN role and implement, communicate, and disseminate supportive policies to ensure a positive practice environment (Bakerjan 2022). Key support mechanisms include defined leadership roles, director‐level accountability, mentoring, membership in strategic committees, networking, healthcare‐university links, and administrative support (Elliott 2017). These require a long‐term strategic approach, with sustained commitment and funding at the organisational level.

### Team Level

4.4

A recent systematic review identified an optimal practice environment as a key enabler of APNs' leadership capacity and impact on health outcomes. This includes positive, supportive relationships with team members, including physicians and administrators, reinforcing the importance of collaborative multidisciplinary practice (Schirle et al. 2020). This is particularly crucial for sole practitioners, as the lone APN role has been shown to limit access to continuing professional development, a concern echoed by APNs in a qualitative research, where they found themselves in ‘quite an isolated role’ (Ryder et al. 2019). Given that advanced practice nursing roles encompass the four pillars of clinical practice, leadership, research, and education, access to continuing professional development is crucial for understanding one's scope of practice and contribution to patient outcomes (Casey et al. 2019). While APNs are often confident in their ability to provide high standards of care, transitioning to advanced practice can lead to uncertainty and apprehension for some (Murphy and Mortimore 2020). Research has linked improved role performance to consistent clinical governance, continuing professional development opportunities, ongoing mentorship, and clear career progression pathways (Evans et al. 2021).

### Advanced Practice Nurse Level

4.5

This review strongly suggests that personal characteristics and education levels play a key role in APN leadership, consistent with other studies (Elliott et al. 2016; Van Kraaij et al. 2020). A qualitative study found that self‐efficacy greatly influenced APNs' confidence and vision for leadership roles (Van Kraaij et al. 2020). Historically, advanced practitioners have come from varied educational backgrounds and training routes, resulting in differences in skills, knowledge, and confidence even among those with similar titles or from the same discipline (Evans et al. 2021). Nonetheless, a shared sense of self‐belief drives individuals within these roles, highlighting the need for structured support mechanisms to develop and strengthen leadership capacity, ultimately enhancing job satisfaction and professional retention. Leadership is not innate but must be developed, practised, and coached, as education alone is insufficient (Lamb et al. 2018). Career position and grade have been identified as stronger predictors of APN leadership activity than education level, underscoring the importance of clear career progression pathways (Sevilla Guerra et al. 2018). Furthermore, longevity in the role allows APNs to draw on extensive clinical experience, a factor often more influential than formal qualifications in shaping leadership effectiveness (Fothergill et al. 2022; McCarthy et al. 2019).

Additionally, self‐leadership, an internal skill that grows with experience, has been shown to improve work performance, support well‐being, and thrive in favourable work environments. A recent review further confirms the positive connection between self‐leadership, work performance, and professional longevity, advocating for its integration into nursing education and continued professional development (Pursio et al. 2025). Furthermore, it underscores the ongoing need for continuous professional development, coaching, and mentorship, aligning with the broader commitment to driving and sustaining progress that ensures nurses remain influential and contribute to the effectiveness of health and social care systems (WHO 2020). Addressing these factors is crucial for optimising leadership potential within advanced practice nursing.

### Strengths and Limitations of the Systematic Review

4.6

This review is strengthened by its adherence to the PRISMA 2020 guidelines (Page et al. 2021), ensuring a clear and reproducible search strategy. The narrative synthesis is based on 14 high‐quality peer‐reviewed studies examining barriers and enablers that influence APNs from enacting leadership. Additionally, the inclusion of studies from four continents provides a broad international perspective and relevance. The earlier review by Elliott et al. (2016) provided a validated framework for categorising factors that influence leadership enactment across four structural levels.

Limitations of this review include the variety of terms associated with the APN role, which complicated the literature search. To mitigate this, multiple search terms were applied across each relevant database. Restricting the search to English‐language publications may have also excluded relevant studies. Additionally, the review was unable to provide the exact number of participants, as one study did not specify the number of APNs. Furthermore, only quantitative studies were included to maintain methodological homogeneity; however, this excluded qualitative and mixed‐methods research, which could have provided valuable insights into leadership experiences. Future research should incorporate a broader range of methodologies to capture the full scope of leadership within advanced practice nursing roles. Finally, due to the heterogeneity of the studies, accurate measurements of leadership could not be obtained. Further research using validated measures of APN leadership capabilities is warranted.

## Implications for Nursing and Health Policy

5

Despite international guidelines on advanced practice nursing (ICN 2020), this review highlights gaps in system‐ and organisational‐level support needed to build the leadership capacity for APNs, ultimately limiting their impact on nursing and health policy. Research suggests that senior decision‐makers often rely on APNs themselves for information about their roles, reinforcing the need for APNs to effectively articulate their leadership contributions (Lamb et al. 2018). APNs must be equipped with the language and tools to demonstrate their leadership impact. The Advanced Practice Nursing Leadership Capabilities Model (Lamb et al. 2018) provides a structured framework to support this by categorising APN leadership into two key domains: ‘patient‐focused leadership’, which involves direct patient care, advocacy, and education, and ‘organization/system‐focused leadership’, which encompasses professional practice development, quality improvement, collaboration, and mentorship. By clearly defining these leadership dimensions, the model helps APNs articulate their contributions more effectively, strengthening their role within healthcare systems and policy development.

Additionally, evidence from Europe suggests that defining advanced practice nursing roles from a service delivery or patient‐care perspective, rather than purely as professional development structures, strengthens the case for both role implementation and sustained integration into healthcare systems (Unsworth et al. 2024). Professional nursing must enhance engagement with policymakers, ensuring that advanced practice nursing roles are not perceived as mere role substitution, but rather as value‐added contributions across diverse healthcare settings (Unsworth et al. 2024). Maximising the leadership potential of APNs can drive clinical practice improvements, service innovation, and long‐term sustainability. Strengthening health systems to support APNs in disease prevention, management, and health promotion is critical, given their advanced training and frontline role in care delivery. Educational preparation is a key factor influencing APNs’ leadership at multiple levels. Variations in educational pathways lead to differences in skills, knowledge, and confidence, highlighting the urgent need for standardised training and master's‐level education, as recommended by the International Council of Nurses (ICN 2020). However, in recognition of global disparities, transitional programmes and bridging courses should be developed to facilitate progression towards this standard.

Building effective leadership capacity for APNs requires a strategic, long‐term approach with sustained institutional support and investment beyond the initial role implementation (Elliott 2017). Leadership development opportunities, such as communities of practice, mentorship, and structured supervision, are essential. APNs in strategic leadership roles can influence long‐term healthcare planning, enhance service delivery, and strengthen the collective nursing voice through collaboration and representation in key decision‐making structures across healthcare settings (Elliott 2017; Rasmussen et al. 2022).

## Conclusion

6

This review provides an evidence‐based update of the factors that influence the enactment of leadership capabilities within advanced practice nursing roles across four structural levels. While findings align with the earlier review by Elliott et al. (2016), this review identifies additional dimensions, such as commitment to leadership capacity building and perceptions of nursing roles, both internal and external to the APN's control. It highlights the ongoing lack of clarity around the APN role, insufficient progress in leadership capacity building at the organisational level, and its impact on how nursing engages with policymakers. The review emphasises the need for leadership education, training, and opportunities to support APNs. Senior decision‐makers at both health system and organisational levels, including nurse leaders, can use these findings to better harness the full potential of APNs to improve system, organisational, and patient outcomes.

## Author Contributions

RF and SL conceived the idea and designed the methodology for this systematic review; RF conducted the search, screened the articles, and extracted the data for write‐up; SL blind‐screened abstracts and full‐text records; SL provided strategic guidance for conducting the review; SL contributed to the data analysis and revised the manuscript; SA and SL approved the final manuscript.

## Conflicts of Interest

The authors declare no conflict of interest.

## References

## Data Availability

The datasets used and/or analysed during the current study are available from the corresponding author on reasonable request.
